# Improvement of thermal stability of highly active species on SiO_2_ supported copper-ceria catalysts

**DOI:** 10.1039/d1ra06204b

**Published:** 2021-10-11

**Authors:** Gonzalo Aguila, Rafael Calle, Sichem Guerrero, Patricio Baeza, Paulo Araya

**Affiliations:** Departamento de Ciencias de la Ingeniería, Facultad de Ingeniería, Universidad Andres Bello Antonio Varas 880 Santiago Chile gonzalo.aguila@unab.cl; Facultad de Ingeniería y Ciencias Aplicadas, Universidad de los Andes, Monseñor Álvaro del Portillo Las Condes Santiago 12455 Chile; Instituto de Química, Pontificia Universidad Católica de Valparaíso Casilla 4059 Valparaíso Chile; Departamento de Ingeniería Química, Biotecnología y Materiales, Facultad de Ciencias Físicas y Matemáticas, Universidad de Chile Beauchef 851 Santiago Chile

## Abstract

CuO–CeO_2_/SiO_2_ catalysts lose activity when they are calcined at 600 °C and temperatures above. This loss of activity was related to a decrease in the amount of highly dispersed Cu species interacting with Ce (CuO–CeO_2_ interface) over the SiO_2_ support. These species are highly active in CO oxidation, so this reaction was selected to conduct this study. In order to avoid the activity loss in CuO–CeO_2_/SiO_2_ catalysts, the effect of high Ce loads (8, 16, 24, and 36%) on the thermal stability of these catalysts was studied. The results reveal that when increasing calcination temperature from 500 to 700 °C, the catalysts with Ce load equal to or higher than 24% increase the formation of highly dispersed Cu interacting with Ce and therefore the activity (90% of CO conversion at 120 °C). In catalysts with Ce load below 24%, Cu species agglomerate and decrease the activity (less than 5% of CO conversion at 120 °C).

## Abstract 2

CuO–CeO_2_/SiO_2_ catalysts with Ce loading of 24% and above keep high activity after calcination at 700 °C. Therefore, a catalyst with high thermal stability of CuO–CeO_2_ interface can be obtained able to work in a higher range of temperatures.

## Graphical abstract

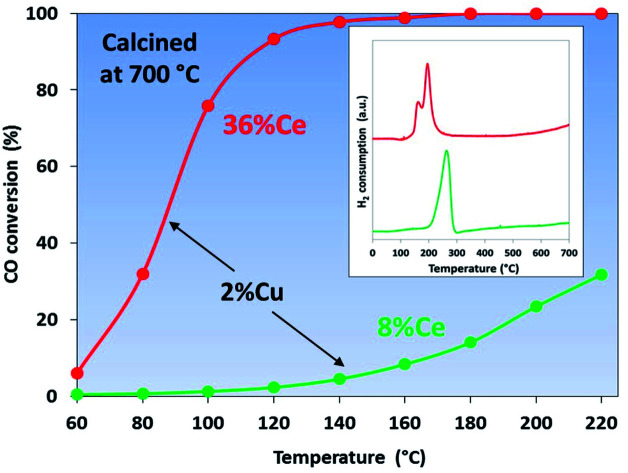

## Introduction

Since the pioneering work of the Flytzani-Stephanopoulos group,^1–3^ who showed the high activity of the CuO–CeO_2_ system in the oxidation reaction of CO with O_2_, this system has also been shown to be highly active in other reactions such as the Water Gas-Shift (WGS) reaction,^4^ and more recently the decomposition of N_2_O.^5–8^

In all these reactions, the interfacial CuO–CeO_2_ sites are responsible for the high activity of these catalysts. Thus, Martinez-Arias *et al.*^9^ studying the oxidation of CO, propose that CO will react with oxygen in the CuO–CeO_2_ interface to produce CO_2_. The controversy on the exact nature of the active sites, the oxidation state of copper, and the reaction mechanism continues.^10,11^ In the case of the WGS, the redox mechanism proposed in the literature assumes that the oxygen of the H_2_O molecule is abstracted in an oxygen vacancy at the CuO–CeO_2_ interface releasing H_2_. The oxygen atom is then removed by CO, allowing the continuation of the catalytic cycle.^4–12^ Finally, the decomposition of N_2_O would occur by the abstraction of the oxygen atom in a vacancy site (Cu^1+^) of the CuO–CeO_2_ interface.^5^ It seems that the higher the dispersion of copper and ceria, the higher the interaction of both metals, which in turn favors the catalytic activity.^13^

Most of the work published in the literature, deals with CuO/CeO_2_ supported catalysts or CuO–CeO_2_ mixed oxides. Some works have been published using Cu and Ce supported on traditional supports like SiO_2_ or alumina^14–21^ in these reactions, and the nature and activity of the CuO–CeO_2_ interface generated over these support is less known, although some works point to the role of labile oxygen species, surface defects, and oxygen vacancies.^14,15,22–29^ Several works dealing with the CuO–CeO_2_ supported catalysts have been done in our laboratory.^19–21^ The oxidizing activity of CO with O_2_ of 2% Cu and 8% Ce systems supported on SiO_2_, Al_2_O_3_, and ZrO_2_ was compared and we found that the most active system was generated by using SiO_2_ as support.^19^ The greater activity of the CuO–CeO_2_/SiO_2_ catalyst was attributed to the fact that the inert character of the silica would favor the formation of the CuO–CeO_2_ interface and thus, improving the interaction between both phases.^16,22,23,25,26,28–30^ Considering the high activity of CuO–CeO_2_ supported on SiO_2_ catalysts, the optimum Cu/Ce load ratio and the total Cu + Ce load of catalysts supported on SiO_2_ were studied in a second work of our group.^20^ The results showed that the optimum Cu/Ce load ratio of catalysts supported on SiO_2_ is very similar to that used by Park *et al.*^18^ for catalysts supported on alumina. In a latter work, Luo *et al.*^17^ found the same optimum of Ce content for Cu–Ce supported on SiO_2_ catalysts calcined at 500 °C. In a third work we studied the thermal stability of catalysts with constant loading of 2% Cu and 8% Ce supported on SiO_2_ changing the calcination temperature in the range of 500–700 °C.^21^ It was found that calcining the catalyst at temperatures from 600 °C generated a noticeable loss of activity and almost a complete deactivation after calcining at 700 °C. Characterization studies on the catalyst calcined at 700 °C showed that the CuO–CeO_2_ interface had decreased substantially due to the migration and segregation of the CuO and CeO_2_ particles on the surface of the silica.^21^

With the purpose of improving the thermal stability of highly dispersed Cu species interacting with Ce (CuO–CeO_2_ interface) over SiO_2_ support, the present work studies the effect of increasing the Ce load above the 8% used in our previous work,^21^ maintaining the Cu load constant at 2%. The hypothesis is that as the Cu species migrate due to the temperature increase, the existence of larger regions of the support covered with CeO_2_ should increase the possibility of contact between CuO and CeO_2_ on the surface of the silica. The oxidation of CO at low temperature has been selected as a test reaction because it is highly sensitive to the formation of the CuO–CeO_2_ interface. Obviously, this reaction does not require high temperatures, so a study of the thermal stability at temperatures above 500 °C does not seem necessary. However, in other reactions like the decomposition of N_2_O, the catalyst must resist reaction temperatures between 600 °C (tail process option) and 850 °C (gas process option),^31^ justifying this study of the thermal stability of CuO–CeO_2_ interface on SiO_2_ support.

## Results and discussion

The specific surface area (SSA) of the catalysts is shown in Table 1, where it is seen that, at each calcination temperature, the SSA decreases as the Ce content increases. This effect is more pronounced at 900 °C, where the SSA of the catalysts decreases from 110 m^2^ g^−1^ (2Cu–8Ce-900 catalyst) to 80 m^2^ g^−1^ (2Cu–36Ce-900 catalyst). However, due to the high thermal stability of silica support (nominal SSA of 130 m^2^ g^−1^), the SSA of the catalysts are relatively high in all the samples, so it seems reasonable to assume that this is not a key parameter in the possible variations of the CuO–CeO_2_ interface.

**Table 1:** Specific surface area, ratio of experimental H_2_ consumed per theoretical H_2_ necessary for total CuO reduction (H_2_ consumption ratio), and CeO_2_ particle size estimated from XRD diffractograms of the CuO–CeO_2_/SiO_2_ catalysts

Catalyst	Specific surface area (m^2^ g^−1^)	H_2_ consumption ratio	CeO_2_ particle size (Å)
2Cu–8Ce-500	128	1.43	58
2Cu–16Ce-500	127	1.66	65
2Cu–24Ce-500	111	1.79	73
2Cu–36Ce-500	108	1.87	94
2Cu–8Ce-700	120	1.16	58
2Cu–16Ce-700	111	1.29	73
2Cu–24Ce-700	119	1.24	97
2Cu–36Ce-700	115	1.44	113
2Cu–8Ce-900	110	0.99	97
2Cu–16Ce-900	91	1.04	117
2Cu–24Ce-900	83	1.03	146
2Cu–36Ce-900	80	0.98	219

**Fig. 1:**
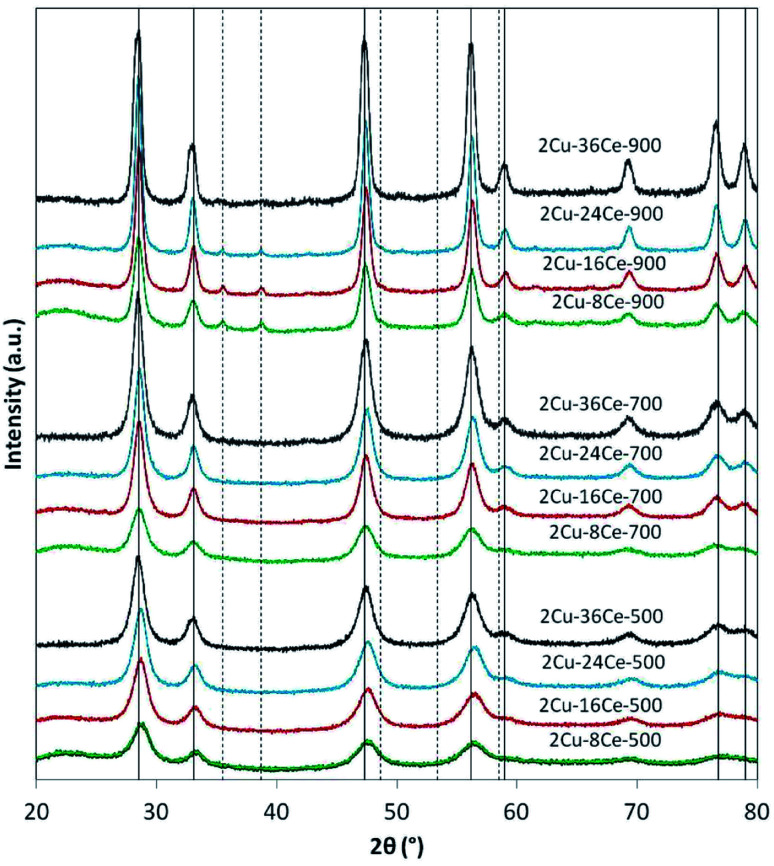
XRD diffractograms of bimetallic catalysts with a constant Cu load (2%) and variable Ce load (8%, 16%, 24% and 36%), calcined at 500, 700 and 900 °C. The nomenclature indicates the Ce loading and the calcination temperature of each catalyst. The characteristics diffraction lines of CuO ( − − ) and CeO_2_ (—) are indicated as reference. Operation conditions: scan range = 20–80°; scan rate = 0.02 degrees per minute.

The XRD analyses of the different samples are shown in Fig. 1. In the catalysts calcined at 500 °C and 700 °C, independently of Ce load, only the peaks of the fluorite phase of CeO_2_ are seen (JCPDS Card N° 81-0792). It is not possible to see the peaks of CuO, so it can be assumed that the Cu phase is highly dispersed at those temperatures. When the catalysts are calcined at 900 °C, the peaks of CuO at 35.5° and 38.7° (JCPDS Card N° 45-0937) are visible in all the catalysts, reflecting the sintering of the Cu species. The intensity of CuO peaks decreases as Ce content increases due to the diluting effect of Ce. Table 1 reports the diameters of CeO_2_ crystalline particles calculated by means of the Scherrer equation, showing that, as expected, at each calcination temperature, the size of the CeO_2_ particles increases as the Ce loading increases. This effect is clearly more important at higher temperatures. In fact, at 900 °C, the CeO_2_ particle diameter increases almost 225% when Ce load increases from 8% to 36%, while at 500 °C, the increment in particle size is only of 162%.

**Fig. 2:**
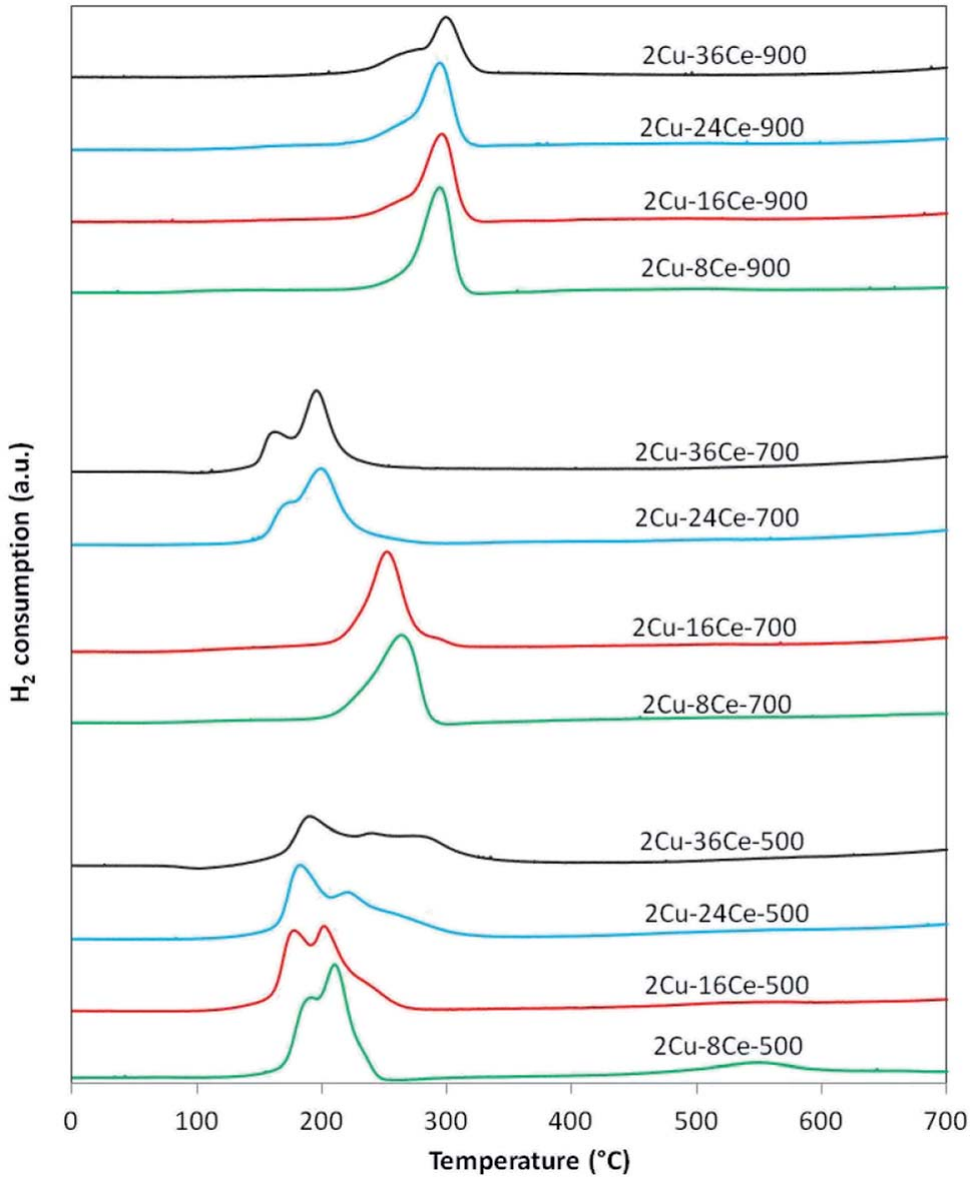
TPR curves of bimetallic catalysts with a constant Cu load (2%) and variable Ce load (8%, 16%, 24% and 36%), calcined at 500, 700 and 900 °C. Experimental conditions: temperature ramp rate: 10 °C min^−1^, flow rate: 20 cm^3^ min^−1^ of 5% H_2_/Ar.

The TPR curves are shown in Fig. 2 and the ratios of the experimental consumption of H_2_ with respect to the theoretical consumption needed to reduce only the CuO to Cu^0^ are presented in Table 1. As shown in Fig. 2, in the case of 2Cu–8Ce-500 and 2Cu–16Ce-500 catalysts it can be observed two reduction peaks, one with a maximum below 200 °C, attributable to the reduction of highly dispersed Cu species in contact with CeO_2_, and a second peak with a maximum between 200 and 225 °C that can be attributed to larger cluster-type Cu species also in contact with CeO_2_.^20^ As the Ce load increases from 8% to 16%, the first reduction peak moves slightly to lower temperature, and the second peak reduce its intensity, indicating the formation of higher amount of highly dispersed Cu species in contact with Ce. For the 2Cu–24Ce-500 catalyst, a third peak appears as a shoulder at 260 °C. The same peak moves to 280 °C for the 2Cu–36Ce-500 catalyst and it is also more intense. This third peak could be attributed to CuO particles interacting with CeO_2_ having higher particle size (see Table 1). These CuO species are poorly crystalline or of small size, as they are not detected by XRD. This is an unexpected result, considering that it is well known that CeO_2_ favors the dispersion of Cu species.^19^ A more deeply study will be performed to elucidate the nature of this CuO species formed at high Ce loading, but as previously noted, it could be related to the growing of CeO_2_ particle size, which decreases the contact between Cu species and CeO_2_. The H_2_ consumption ratio of catalysts calcined at 500 °C is always superior to 1, indicating an important reduction of CeO_2_ in contact with Cu species. Calcination at 700 °C produces a different effect in Cu dispersion depending on Ce content. In fact, for the catalysts with 8% and 16% of Ce content (2Cu–8Ce-700 and 2Cu–16Ce-700), the low temperature peaks disappears, and only the reduction peak associated with the reduction of CuO species in contact with CeO_2_ can be observed in the range between 260–280 °C, thus reflecting the agglomeration of dispersed Cu species. In contrast, the TPR curves of the catalysts with the highest Ce load (2Cu–24Ce-700 and 2Cu–36Ce-700), the high temperature peaks disappears and only peaks with maxima below 200 °C can be observed. In other words, the Cu species migrate and disperse forming highly dispersed CuO species in contact with CeO_2_. Finally, when calcining at 900 °C, all the catalysts show a main reduction peak close to 300 °C, characteristic of crystalline CuO species or bulk CuO poorly or not interacting with CeO_2_. Also, the H_2_ consumption ratio of catalysts calcined at 900 °C is almost 1 in all cases, indicating no reduction of CeO_2_, *i.e.*, the CuO–CeO_2_ interface disappears.

**Fig. 3:**
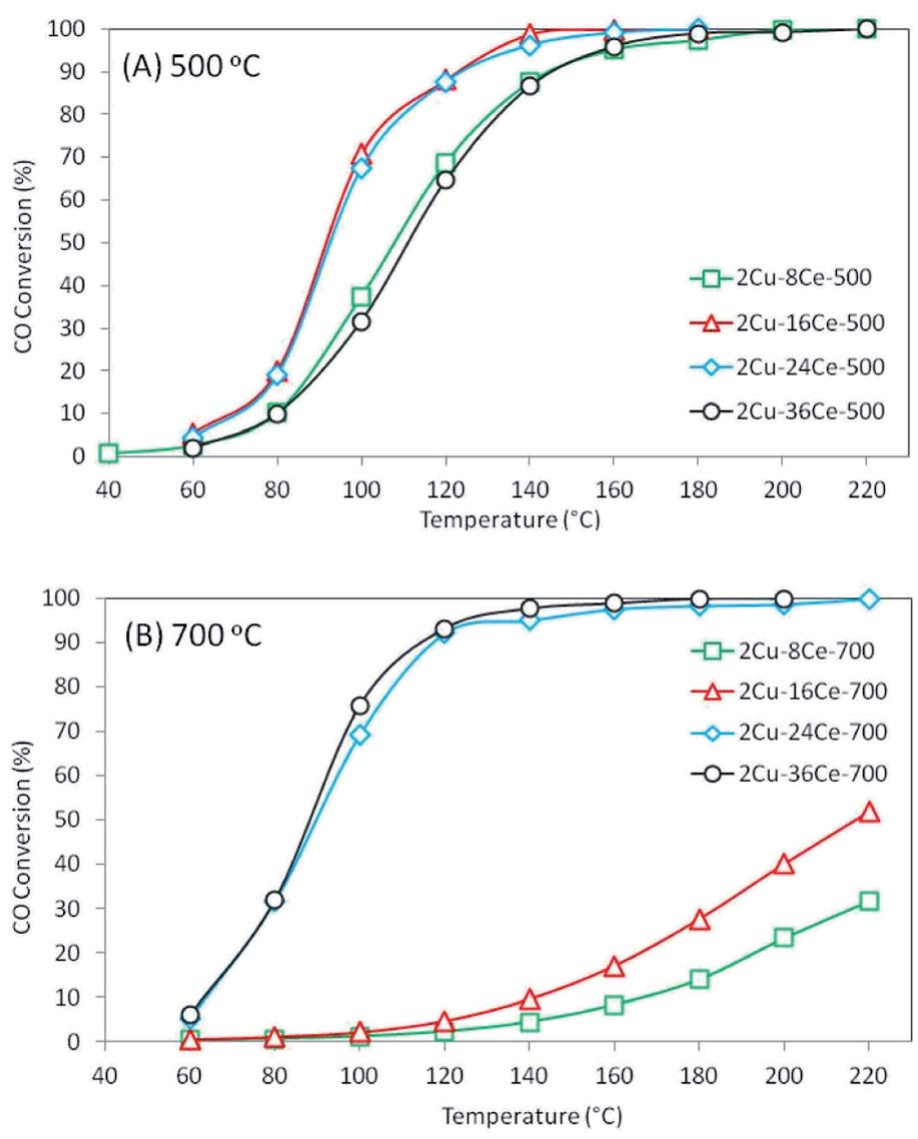
CO conversion curves of bimetallic catalysts with a constant Cu load (2%) and variable Ce load (8%, 16%, 24% and 36%) calcined at 500 °C and 700 °C. (A) Catalysts calcined at 500 °C: 2Cu–8Ce-500 (green); 2Cu–16Ce-500 (red); 2Cu–24Ce-500 (blue); 2Cu–36Ce-500 (black). (B) Catalysts calcined at 700 °C: 2Cu–8Ce-700 (green); 2Cu–16Ce-700 (red); 2Cu–24Ce-700 (blue); 2Cu–36Ce-700 (black). Reaction conditions: temperature ramp rate = 3 °C min^−1^, CO concentration = 2% v/v, O_2_ concentration = 3% v/v, mass of catalysts = 0.1 gram, total flow rate = 100 cm^3^ min^−1^.

The results of the conversion of CO with temperature for the different catalysts are shown in Fig. 3. As can be seen in Fig. 3(A), when catalysts are calcined at 500 °C, the activity increases as the Ce content is increased from 8% to 16%, and the activity remains constant when the Ce content is raised to 24%. However, increasing the Ce loading to 36% led to a decrease in activity, which shows the existence of an optimum Ce loading (16% Ce) in catalysts with 2% Cu loading and calcined at 500 °C, and this result is in agreement with a previous study of our group.^20^ The behavior of these catalysts can be explained by the activity of the different copper species formed on the surface. As discussed in the literature, the highly dispersed Cu species in contact with Ce are the most active species of the CuO–CeO_2_ system,^32^ while the bulk CuO species is practically inactive at temperatures below 200 °C.^19^ Therefore, the increase of activity as Ce loading increases from 8% to 16% is in agreement with the increase in the concentration of highly dispersed Cu species in contact with CeO_2_ observed in the TPR experiment. The concentration and reducibility of the highly dispersed Cu species in contact with CeO_2_ (first peak below 200 °C) on the 2Cu–24Ce-500 catalyst are similar to those of the 2Cu–16Ce-500 catalyst, and this explains why their activities are also very similar. When the Ce content increases to 36%, the TPR curve of the 2Cu–36Ce-500 catalyst clearly shows a decrease in the amount of highly dispersed Cu species in contact with CeO_2_ and an increase of CuO in contact with CeO_2_, explaining the lower activity of the 2Cu–36Ce-500 catalyst compared to that of the 2Cu–24Ce-500 catalyst.

When catalysts were calcined at 700 °C, as shown in Fig. 3(B), the 2Cu–8Ce-700 and 2Cu–16Ce-700 catalysts undergo a substantial loss of activity respect to homologous catalysts calcined at 500 °C (Fig. 3(A)). The TPR curves explain this activity decrease due to the agglomeration of Cu species in contact with CeO_2_, with the resulting loss of the amount of highly dispersed Cu species. In contrast, the 2Cu–24Ce-700 and 2Cu–36Ce-700 catalysts show very high and similar activities after calcination at 700 °C. In fact, the 2Cu–24Ce-700 catalyst retains almost the same activity to that found when calcined at 500 °C, while the 2Cu–36Ce-700 catalyst substantially increases its activity. The TPR curves of both catalysts, 2Cu–24Ce-700 and 2Cu–36Ce-700, show an increase in the concentration of highly dispersed Cu species in contact with CeO_2_, while CuO particles interacting with high particle size CeO_2_ disappears, which explains the very high activity of these catalyst. It is important to note that in these catalysts calcined at 700 °C, the optimum Ce loading is 24%, showing that optimum Ce loads depend on the calcination temperature.

Finally, by calcining at 900 °C, none of the catalysts is active below 200 °C (CO conversion curves were not included) and this is accounted for by the strong sintering and segregation of the CuO and CeO_2_ species, destroying the active CuO–CeO_2_ interface and forming mainly CuO crystalline species or bulk CuO, as seen in the TPRs and XRDs of these catalysts. The above is related to that H_2_ consumption ratios are almost equal to 1 (see Table 1) in these catalysts calcined at 900 °C, indicating that CeO_2_ reduction does not occur.

## Experimental

### Catalyst preparation

The catalysts were prepared by coimpregnation of SiO_2_ support (Aerosil 130, Degussa). This is a non-porous, amorphous, hydrophilic fumed silica of 99.8% of SiO_2_ content having a specific surface area of 130 m^2^ g^−1^, with traces of impurities of metal in the ppb range. This support was coimpregnated with an aqueous solution containing the proper amounts of both Cu and Ce nitrates, to obtain a constant load of 2% Cu and variable loads of 8%, 16%, 24%, and 36% (w/w) of Ce. The catalysts were dried in an oven at 105 °C for 12 hours. Each catalyst was separated into three samples and calcined in a muffle furnace at 500, 700 and 900 °C for 3 hours under air. The catalysts were identified as 2Cu-XCe-*Y*, where X indicates the Ce load, and Y is the calcination temperature.

### Catalyst characterization

The samples were characterized by N_2_ adsorption, X-ray diffraction (XRD), and temperature programmed reduction (TPR) in a hydrogen stream.

Determination of the BET specific surface area of the catalysts was made by obtaining the N_2_ adsorption and desorption isotherms in a Micromeritics equipment, Model ASAP 2010 Sorptometer. The samples were previously degassed at 200 °C.

The crystal structure of the different catalysts was determined on a Siemens D-5000 diffractometer using Cu Kα radiation and a scan rate of 0.02 degrees per minute.

Finally, the temperature programmed reduction analyses were made on a conventional system equipped with a TCD detector, with a flow rate of 20 cm^3^ min^−1^ of a gaseous mixture of 5% H_2_ in Ar, at a heating rate of 10 °C min^−1^ between ambient temperature and 700 °C.

### Measurement of catalyst activity

The kinetics tests were made in a piston flow tubular reactor, with a stream of 2% CO and 3% O_2_ at a total flow rate of 100 cm^3^ min^−1^. After loading the reactor with 0.1 g of catalyst, the sample was pretreated at 300 °C for one hour in O_2_, and the reactor was cooled down to room temperature. The reactants were then fed, and the temperature was increased at a rate of 3 °C min^−1^, taking samples every 20 °C to determine the concentration of CO, O_2_, and CO_2_, using a PerkinElmer Autosystem Chromatograph with a CTR column (Alltech) and a TCD detector.

## Conclusions

This work shows that Ce content is a crucial factor to determine the formation of different Cu species with the calcination temperature, in the range 500–700 °C. At Ce content below 24%, the Cu species agglomerates after calcination at 700 °C, decreasing the amount of highly disperse Cu species interacting with CeO_2_ (CuO–CeO_2_ interface) leading to low activity in these catalysts. At Ce content of 24% or higher, calcination at 700 °C increases Cu dispersion and the amount of highly disperse CuO–CeO_2_ species, thus increasing catalysts activity.

These new findings open the possibility of synthesizing CuO–CeO_2_/SiO_2_ catalysts with a high thermal stability of CuO–CeO_2_ interface over a wider temperature range, allowing its use in reactions that require high temperatures.

## Author contributions

Gonzalo Aguila: conceptualization, methodology, investigation, formal analysis, validation, visualization, writing – original draft. Rafael Calle: initial research, experimental work. Sichem Guerrero: formal analysis, investigation, validation, writing – review & editing. Patricio Baeza: formal analysis, validation, visualization, writing – original draft. Paulo Araya: conceptualization, funding acquisition, project administration, resources, supervision, writing – review & editing.

## Conflicts of interest

There are no conflicts to declare.

## Supplementary Material
